# A Unique Case of Aeromonas salmonicida Peritonitis in a Patient Undergoing Peritoneal Dialysis in Mexico

**DOI:** 10.7759/cureus.90226

**Published:** 2025-08-16

**Authors:** Raúl Romero Feregrino, Daniel O Pacheco Rosas, Arantxa Juárez Castillo, Melissa Takashima Castro, Juan M Ruíz Ruíz

**Affiliations:** 1 Pediatrics, Mexican Academy of Pediatrics, Mexico City, MEX; 2 Pediatrics/Infectious Diseases Service, Hospital de Pediatría Centro Médico Nacional (CMN) Siglo XXI, Mexico City, MEX; 3 Pediatrics, Hospital de Pediatría Centro Médico Nacional (CMN) Siglo XXI, Mexico City, MEX; 4 Microbiology Lab, Hospital de Pediatría Centro Médico Nacional (CMN) Siglo XXI, Mexico City, MEX

**Keywords:** aeromonas salmonicida, pediatric patient, peritoneal dialysis, peritonitis, rare infection

## Abstract

*Aeromonas salmonicida* is a Gram-negative, oxidase-positive, facultative anaerobic bacterium primarily recognized as a pathogen in fish. Its isolation in humans is rare and scarcely reported, particularly in cases of peritonitis. We present the case of a 16-year-old female patient with end-stage chronic kidney disease, recent renal graft rejection, and undergoing continuous ambulatory peritoneal dialysis, who presented with severe abdominal pain and cloudy peritoneal effluent. Fluid analysis revealed an elevated leukocyte count (15,022 cells/μL, including 13,330 polymorphonuclear cells). Empirical treatment with cefalotin and amikacin was initiated. Following the observation of short Gram-negative bacilli, the regimen was adjusted to intraperitoneal cefepime and amikacin. After 72 hours, the VITEK® 2 automated system identified *A. salmonicida* with 98% probability. The patient showed clinical and laboratory improvement after 14 days of targeted therapy. Human infections caused by *A. salmonicida* are exceptionally rare. This case highlights the importance of considering atypical pathogens in immunocompromised patients with peritonitis. Given the challenges in its identification, the use of automated or molecular diagnostic methods is recommended. Although no clear aquatic exposure was identified, the isolation of this microorganism supports the need for broad microbiological surveillance. Antimicrobial susceptibility profiles should be evaluated on a case-by-case basis.

## Introduction

Bacterial peritonitis remains a significant cause of morbidity in patients with chronic conditions, such as kidney failure requiring peritoneal dialysis, as well as in those with advanced liver disease [1]. Although the most frequent pathogens are enteric bacteria and Gram-positive cocci, there are rare reports of infections caused by environmental microorganisms, particularly those associated with aquatic environments [2].

Among these atypical pathogens is *Aeromonas salmonicida*, a Gram-negative, oxidase-positive, facultative anaerobic bacillus, primarily known for its role in infectious diseases of freshwater fish [3], where it is the main cause of furunculosis in various species. In addition to water, *Aeromonas* species are distributed in diverse sources, such as soil, vegetables, and food. Human infection by *A. salmonicida* is extremely rare, especially compared to other species of the genus, such as *A. hydrophila* or *A. caviae*, which have been linked to gastroenteritis, skin infections, and bacteremia [4].

This report describes a case of peritonitis caused by *A. salmonicida* in a pediatric patient, highlighting its microbiological characteristics and the clinical importance of considering this pathogen in scenarios where it may not typically be identified.

## Case presentation

A 16-year-old female patient from Oaxaca, Mexico, with a history of kidney transplantation in October 2023 and graft rejection in April 2024. She has stage 5 chronic kidney disease, diagnosed at the age of seven, of undetermined cause. Since January 31, 2025, she has been undergoing continuous ambulatory peritoneal dialysis.

She presented with a 10-day history of generalized abdominal pain of moderate intensity (visual analogue scale: 5/10), without associated symptoms or initial functional limitation. The pain showed transient improvement but recurred a week later with increased intensity, reaching a visual analogue scale of 9/10, persistent even at rest, and aggravated by minimal contact. From the onset, the patient noticed cloudiness in the peritoneal fluid during exchanges, with partial improvement that was lost a day prior to admission.

Upon arrival, she reported episodes of intense, intermittent abdominal pain, particularly at night, requiring analgesia with buprenorphine on several occasions. Tachycardia was recorded, associated with pain exacerbations. A nasogastric tube was placed, draining bile-stained contents. The peritoneal fluid obtained was turbid and whitish, with a cell count of 15,045 cells, including 15,022 leukocytes (13,330 polymorphonuclear and 1,692 mononuclear cells). Gram staining did not reveal microorganisms. Empirical treatment with intraperitoneal amikacin and cefalotin was initiated under the clinical diagnosis of peritonitis.

The next day, the laboratory reported the presence of short Gram-negative bacilli in the dialysis fluid. The antibiotic regimen was adjusted to intraperitoneal cefepime and amikacin to cover *Enterobacteriaceae* and *Acinetobacter baumannii *based on the observed morphology.

**Figure 1:**
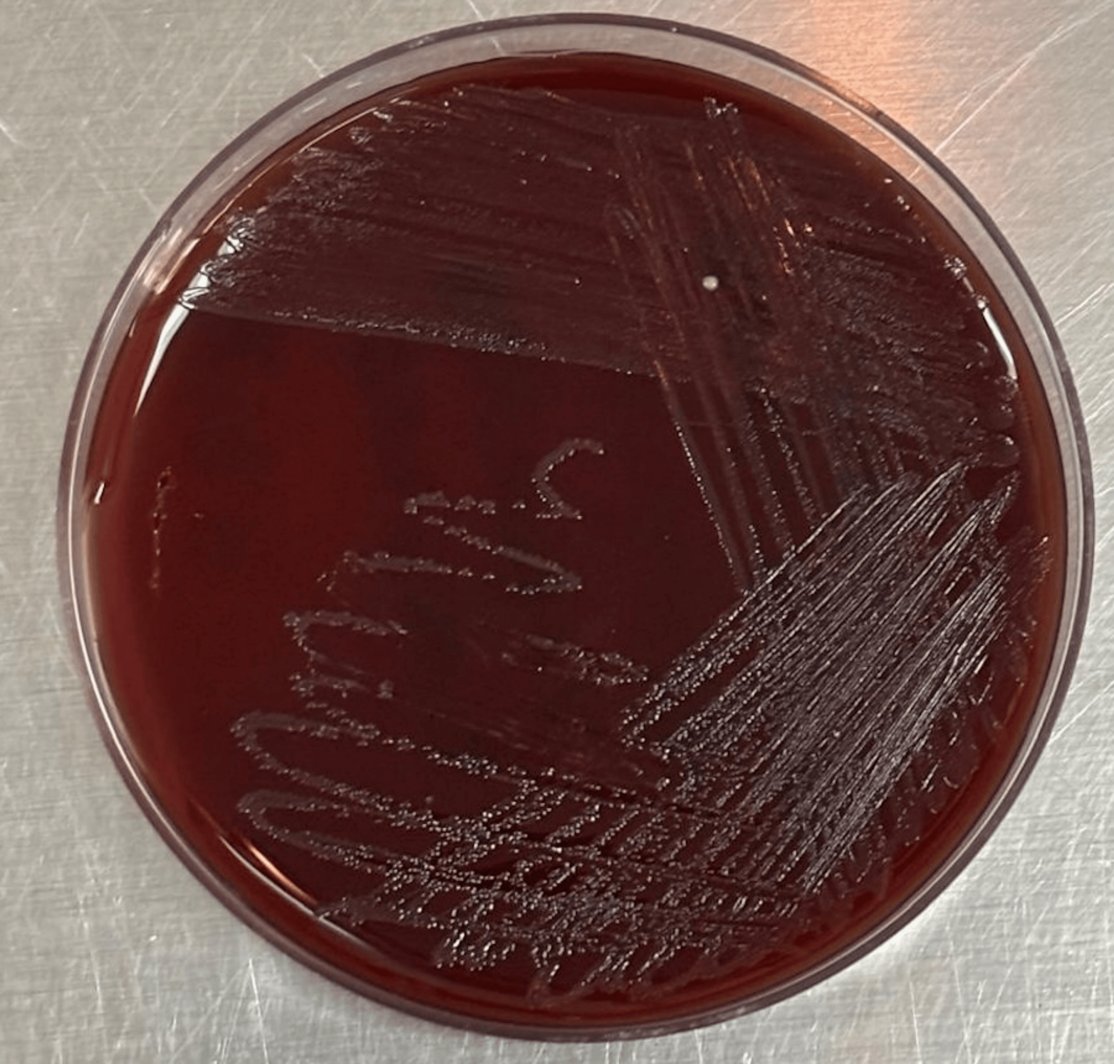
Blood agar medium showing colonies of Aeromonas salmonicida.

After 24 hours of aerobic incubation at 37°C on blood agar, significant growth (≥10^5^ colony-forming units/mL) of small, grayish colonies was observed (Figure 1). Gram staining confirmed Gram-negative bacilli. Oxidase and catalase tests were positive, and no motility was observed. On day three, using the automated VITEK® 2 system (bioMérieux, France), *A. salmonicida* was identified with 98% probability.

Clinically, the patient experienced reduced pain (visual analogue scale: 4) without fever. The antibiotic treatment was continued for 14 days. At the end of therapy, the patient was asymptomatic, and analysis of the peritoneal fluid showed normal cell counts (10 cells, 9 leukocytes). She was discharged and sent home.

## Discussion

Infections caused by *Aeromonas *species in humans have been mainly associated with exposure to aquatic environments or ingestion of contaminated food [5]. Although *A. hydrophila* and other species are more frequently identified, *A. salmonicida* is infrequent, and there is limited clinical information regarding its involvement in abdominal infections [6]. Nevertheless, identification of this bacterium in our case underscores the importance of maintaining broad surveillance in peritonitis of unknown etiology.

The main risk factor associated with *A. salmonicida* infections appears to be exposure to contaminated water sources, either through direct consumption or consumption of fish products. However, no such exposure history was documented in this case [7].

From a microbiological perspective, *A. salmonicida* exhibits unique features: it can show pigmentation; some strains have difficulty growing at 37°C; and, unlike other *Aeromonas* species, it may be non-motile [8]. Its virulence factors include hemolysins, lipases, type III secretion systems, and other molecular elements that facilitate tissue invasion and immune system evasion [9].

In the context of bacterial peritonitis, colonization of the peritoneal fluid by *Aeromonas* mainly occurs via hematogenous spread. Although the colonization mechanism is relatively well known, the exact entry route into the body remains unclear. It has been suggested that contact with aquatic environments or disinfectants, which is common in peritoneal dialysis patients, could be a factor. However, in other cases, such exposure cannot be confirmed. Since the intestinal tract is considered the organic reservoir, bacterial translocation followed by bacteremia has also been proposed as a possible mechanism of infection [10].

Diagnosis of this bacterium may require additional techniques such as mass spectrometry (MALDI-TOF) or genetic sequencing, as traditional phenotypic methods often do not allow accurate identification [11]. Regarding treatment, *A. salmonicida* is usually sensitive to fluoroquinolones and third-generation cephalosporins, although confirming the susceptibility profile through specific testing is essential [12,13].

The importance of reporting this case lies in the scarcity of information on isolates of this species in Mexico; most reports refer to soft tissue infections [14], with no previous descriptions of peritonitis. This case highlights the need not to underestimate the role of uncommon agents in severe infections, especially in immunocompromised patients or those with a history of contact with aquatic environments, and emphasizes the importance of a thorough microbiological approach when initial therapy is ineffective.

Finally, the recent increase in identification of this microorganism may be due to the greater availability of automated identification systems, as well as increased clinical awareness of uncommon pathogens, particularly in immunosuppressed patients.

## Conclusions

*A. salmonicida*, though rarely reported in humans, should be considered a possible etiological agent in peritonitis, particularly in immunocompromised patients or those with a history of peritoneal dialysis, as its identification may require advanced microbiological techniques. The absence of a clear history of exposure to aquatic environments in some cases highlights the need to explore other infection routes, such as intestinal bacterial translocation and bacteremia, to better understand the pathogenesis of *A. salmonicida* infections. The increase in *A. salmonicida* isolation in clinical settings may be related to the implementation of automated methods and increased recognition of uncommon pathogens, reinforcing the importance of detailed microbiological diagnosis to guide appropriate treatment.
